# Respiratory Ammonification of Nitrate Coupled to Anaerobic Oxidation of Elemental Sulfur in Deep-Sea Autotrophic Thermophilic Bacteria

**DOI:** 10.3389/fmicb.2017.00087

**Published:** 2017-01-30

**Authors:** Galina B. Slobodkina, Andrey V. Mardanov, Nikolai V. Ravin, Anastasia A. Frolova, Nikolay A. Chernyh, Elizaveta A. Bonch-Osmolovskaya, Alexander I. Slobodkin

**Affiliations:** ^1^Winogradsky Institute of Microbiology, Research Center of Biotechnology of the Russian Academy of SciencesMoscow, Russia; ^2^Institute of Bioengineering, Research Center of Biotechnology of the Russian Academy of SciencesMoscow, Russia

**Keywords:** respiratory ammonification, nitrate reduction, DNRA, microbial sulfur oxidation, elemental sulfur, deep-sea microorganisms

## Abstract

Respiratory ammonification of nitrate is the microbial process that determines the retention of nitrogen in an ecosystem. To date, sulfur-dependent dissimilatory nitrate reduction to ammonium has been demonstrated only with sulfide as an electron donor. We detected a novel pathway that couples the sulfur and nitrogen cycles. Thermophilic anaerobic bacteria *Thermosulfurimonas dismutans* and *Dissulfuribacter thermophilus*, isolated from deep-sea hydrothermal vents, grew autotrophically with elemental sulfur as an electron donor and nitrate as an electron acceptor producing sulfate and ammonium. The genomes of both bacteria contain a gene cluster that encodes a putative nitrate ammonification enzyme system. Nitrate reduction occurs via a Nap-type complex. The reduction of produced nitrite to ammonium does not proceed via the canonical Nrf system because nitrite reductase NrfA is absent in the genomes of both microorganisms. The genome of *D. thermophilus* encodes a complete sulfate reduction pathway, while the Sox sulfur oxidation system is missing, as shown previously for *T. dismutans*. Thus, in high-temperature environments, nitrate ammonification with elemental sulfur may represent an unrecognized route of primary biomass production. Moreover, the anaerobic oxidation of sulfur compounds coupled to growth has not previously been demonstrated for the members of *Thermodesulfobacteria* or *Deltaproteobacteria*, which were considered exclusively as participants of the reductive branch of the sulfur cycle.

## Introduction

Respiratory ammonification (also known as dissimilatory nitrate reduction to ammonium, DNRA) and denitrification are two main microbial processes of nitrate reduction that determine the retention of nitrogen in an ecosystem. In many natural and anthropogenic environments, biogeochemical cycles of nitrogen and sulfur are coupled due to the activity of autotrophic microorganisms. It is well documented that different phylogenetic groups of prokaryotes use elemental sulfur, sulfur oxyanions, and hydrogen sulfide as electron donors for denitrification (Thamdrup and Dalsgaard, 2008; Shao et al., 2010); however, sulfur-dependent nitrate ammonification (DNRA with oxidation of sulfur compounds) has so far been demonstrated only with sulfide as an electron donor (listed below). (i) Sulfate-reducing bacteria *Desulfovibrio desulfuricans* and *Desulfobulbus propionicus* produce ammonium from nitrate using sulfide, but cannot couple this process with growth (Dannenberg et al., 1992). (ii) Epsilon-proteobacterium *Sulfurospirillum deleyianum* oxidizes sulfide with nitrate during growth, thus producing ammonium and intracellular elemental sulfur, which is not further metabolized (Eisenmann et al., 1995). (iii) Samples of filamentous sulfur gamma-proteobacteria *Beggiatoa* and *Thioploca* form ammonium from nitrate while oxidizing H_2_S to internally stored sulfur (Otte et al., 1999; Sayama et al., 2005; Hogslund et al., 2009); presumably sulfur could be oxidized further, but this has not been directly proven. Moreover, physiological mechanisms of utilization of intracellular sulfur globules and oxidation of extracellular insoluble sulfur could be significantly different (Franz et al., 2009; Berg et al., 2014). Thus, microorganisms that can couple growth with respiratory ammonification of nitrate using elemental sulfur as an electron donor are unknown.

Elemental sulfur is abundant in many types of thermal environments, including deep-sea hydrothermal vents (Nakagawa et al., 2006). Thermophilic anaerobic chemolithoautotrophic bacteria *Thermosulfurimonas dismutans* (phylum *Thermodesulfobacteria*) and *Dissulfuribacter thermophilus* (class *Deltaproteobacteria*) were isolated from deep-sea sulfidic chimney-like deposits as sulfur-disproportionating microorganisms (Slobodkin et al., 2012, 2013). *T. dismutans* and *D. thermophilus* were recovered from different samples collected at the same hydrothermal field (Mariner 1910 m deep, Eastern Lau Spreading Center, Pacific Ocean) and have 13°C difference in their optimal growth temperature (74°C for *T. dismutans* and 61°C for *D. thermophilus*). Both bacteria have similar physiology and are incapable of dissimilatory sulfate reduction and utilization of organic electron donors. In addition to disproportionation of elemental sulfur, thiosulfate, and sulfite, these microorganisms can oxidize molecular hydrogen with thiosulfate as an electron acceptor. Sequencing and analysis of the *T. dismutans* genome revealed pathways for autotrophic carbon and nitrogen fixation, sulfur disproportionation, and nitrate reduction (Mardanov et al., 2016). However, in the original descriptions of *T. dismutans* and *D. thermophilus*, the capacity for anaerobic oxidation of elemental sulfur coupled to nitrate reduction was not tested.

In this report, we present results obtained during further investigations of the metabolic potential of these microorganisms together with data on the sequencing and analysis of the *D. thermophilus* genome. We show that *T. dismutans* and *D. thermophilus* can grow and oxidize elemental sulfur with nitrate as an electron acceptor producing sulfate and ammonium.

## Materials and Methods

### Strains and Cultivation Conditions

*Thermosulfurimonas dismutans* S95^T^ (DSM 24515^T^) and *Dissulfuribacter thermophilus* S69^T^ (DSM 25762^T^) were obtained from the German Collection of Microorganisms and Cell Cultures (Leibniz Institute DSMZ) and were routinely cultivated in anaerobic autotrophic medium with elemental sulfur (purum p.a. > 99.5%, Sigma-Aldrich, 150 mmol l^-1^) as the energy source, ferrihydrite [poorly crystalline Fe(III) oxide; 90 mmol Fe(III) l^-1^] as the sulfide scavenging agent, and ${\text{CO}}_{2}/{\text{HCO}}_{3}^{-}$ (100% CO_2_ in the gas phase) as the sole carbon source. The composition of the medium and the preparation technique is described in detail elsewhere (Slobodkin et al., 2012). pH of the autoclaved medium was 6.7–6.8 (measured at 25°C). The temperature of incubation was 65°C for *T. dismutans* and 60°C for *D. thermophilus*. Both microorganisms grow by elemental sulfur disproportionation only in the presence of ferrihydrite, which chemically reacts with produced sulfide forming insoluble FeS, and therefore decreasing the inhibitory effect of HS^-^. However, at the growth conditions where sulfide is not produced the presence of ferrihydrite seems to be not necessary. In experiments on the utilization of nitrate as an electron acceptor and elemental sulfur as an electron donor, ferrihydrite was omitted from the medium and potassium nitrate (10 mM) was added from a sterile stock solution. Sodium thiosulfate (15 mM), sodium sulfite (5 mM), or sodium sulfide (4 mM) was added to a nitrate-containing medium instead of elemental sulfur in experiments aimed at testing these compounds as potential electron donors. At least three successive 5% (v/v) transfers of each microbial culture were made in the nitrate-containing media to get rid of ferrihydrite before quantitative growth studies. Growth determinations and analyses of all chemical compounds were made in nitrate-containing medium from which ammonium chloride was omitted. All experiments were conducted in 17-ml Hungate tubes filled with 10 ml of the medium. Series of the Hungate tubes were incubated in parallel, and for each measuring point, three independent test tubes were used. All experiments were repeated twice. Mean square deviations for the six measurements are presented throughout the paper.

### Preparation of Elemental Sulfur Separated from Microbial Cells

For the experiments with restricted contact between bacterial cells and elemental sulfur, solid sulfur (purum p.a. > 99.5%, Sigma-Aldrich) was embedded in alginate beads. The technique of preparation of alginate beads was adopted from Gavrilov et al., 2012. A freshly prepared suspension of sulfur in distilled water (100 g l^-1^) was slowly added to 1.5% (w/v) solution of sodium alginate under intense stirring. The suspension was left for 30 min to eliminate air bubbles, then gently stirred for an hour and subsequently manually dripped through a micropipette tip into a solution of 33 g l^-1^ CaCl_2_ from a height of ca. 10 cm. The beads formed were of 2.0–2.5 mm diameter and were left in CaCl_2_ solution overnight to cure, then separated from the solution and washed on a vacuum filter with distilled water. Wet beads were dispensed into Hungate tubes to the bulk volume of ca. 1.5 ml. After adding the beads, the tubes were filled with liquid freshly prepared medium of the same composition as used in growth experiments to the total volume of 10 ml under CO_2_ efflux. The obtained medium contained ca. 150 mmol l^-1^ of elemental sulfur entrapped in alginate. The medium was sterilized by autoclaving at 105°C.

### Analytical Methods

Growth of bacteria was determined by direct counting of the cells with a phase-contrast microscope (Olympus CX41) and a counting chamber. Ammonium was determined by the phenol-hypochlorite reaction (Chaney and Marbach, 1962). Sulfate, sulfite, thiosulfate, nitrate, and nitrite were analyzed by HPLC with a Stayer ion chromatograph (Aquilon) equipped with an IonPack AS4-ASC column (Dionex) and conductivity detector; the eluent was bicarbonate (1.36 mM)/carbonate (1.44 mM) and the flow rate was 1.5 ml min^-1^. Sulfide was measured with dimethyl-*p*-phenylenediamine (Trüper and Schlegel, 1964).

### Sequencing and Annotation of Genomes

The *D. thermophilus* S69^T^ genome was sequenced with a Roche Genome Sequencer (GS FLX) using the Titanium XL+ protocol for a shotgun genome library. About 90 Mb of sequences with an average read length of 411 bp were generated in the GS FLX run. The GS FLX reads were *de novo* assembled using Newbler Assembler version 2.9. The draft genome of *D. thermophilus* S69^T^ was compiled from 27 contigs longer than 500 bp, with a total contig length of 2,524,218 bp.

The genome of *T. dismutans* S95^T^ was sequenced earlier (Mardanov et al., 2016).

Gene calling, annotation, and analysis were performed for all contigs longer than 500 bp. Genes were annotated using the RAST server (Brettin et al., 2015). The annotation was manually corrected using a search against the NCBI sequence databases. The transmembrane helices were predicted with TMHMM Server v. 2.0^1^. The N-terminal signal peptides were predicted using Signal P v.4.1 for Gram-negative bacteria^2^ and PRED-TAT^3^.

### Phylogenetic Analyses of NapA Genes

Amino acid sequences were transferred into the MEGA 6.0 software suite, and aligned using CLUSTAL W. Amino acid sequences of NapA were downloaded from GenBank. The aligned amino acid sequence data were used to construct the phylogenetic trees based on Maximum Likelihood algorithm with 1000 time bootstrap resamplings in MEGA 6.0 software (Tamura et al., 2013).

### Nucleotide Sequence Accession Number

The annotated genome sequence of *D. thermophilus* has been deposited in the GenBank database under accession no. MAGO00000000. The GenBank accession number for genome sequence of *T. dismutans* is LWLG00000000 (Mardanov et al., 2016).

## Results

### Autotrophic Growth with Elemental Sulfur and Nitrate

**FIGURE 1:**
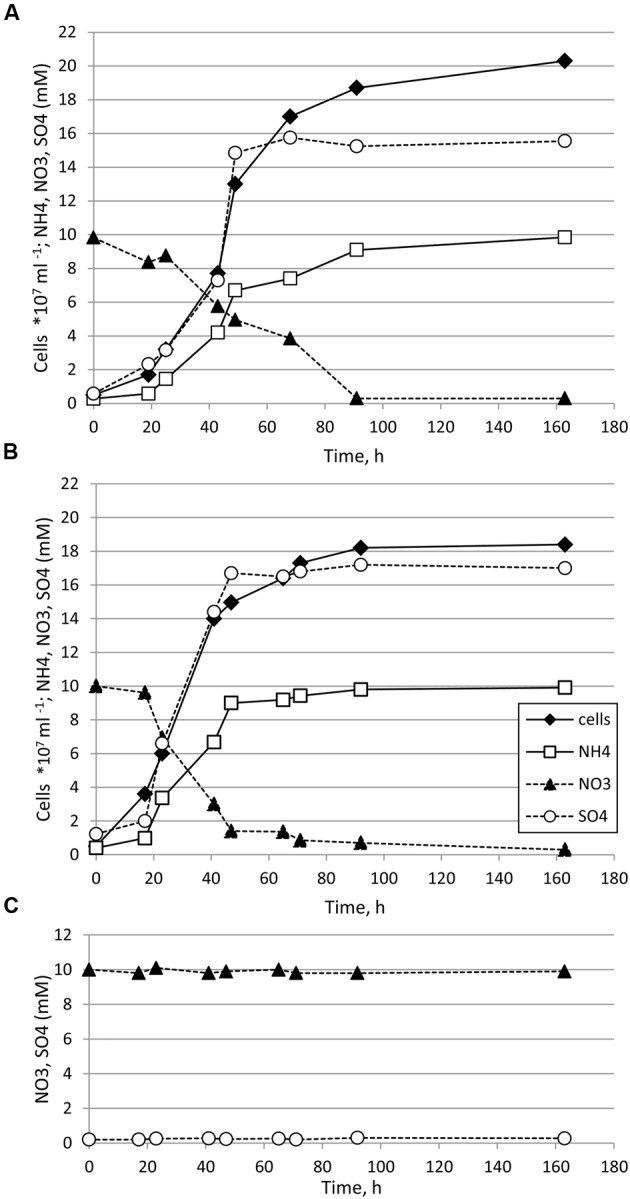
**Growth of *Thermosulfurimonas dismutans* (A)** and *Dissulfuribacter thermophilus* **(B)** in medium with elemental sulfur (oxidized to sulfate), nitrate (reduced to ammonium), and CO_2_/HCO_3^-^_. Initial concentration of elemental sulfur was 150 mmol l^-1^. Concentrations of thiosulfate, sulfite, and nitrite were below the detection limit (ca. 0.01 mM) in all samples. **(C)** In non-inoculated controls concentration of nitrate was not changed, concentration of ammonium was below the detection limit (ca. 0.02 mM) and no sulfate accumulation was detected.

*Thermosulfurimonas dismutans* and *Dissulfuribacter thermophilus* grew in batch cultures in liquid anaerobic cultivation medium supplemented with elemental sulfur as an electron donor and nitrate as an electron acceptor (**Figures 1A,B**). Maximal cell density was 2.0–2.1 × 10^8^ cells ml^-1^ for *T. dismutans* and 1.8–1.9 × 10^8^ cells ml^-1^ for *D. thermophilus*. The specific growth rate of *T. dismutans* was 0.084 ± 0.004 h^-1^ (doubling time: 8.25 h) while the specific growth rate of *D. thermophilus* was 0.115 ± 0.006 h^-1^ (doubling time: 6.03 h). The mode of nitrate and sulfur metabolism was similar for both microorganisms. Cell growth was coupled to the removal of nitrate and the accumulation of ammonium and sulfate. Nitrate was completely reduced to ammonium: from 10.00 ± 0.53 mM of ${\text{NO}}_{3}^{-}$, *T. dismutans* produced 9.85 ± 0.51 mM of ${\text{NH}}_{3}^{+}$ and *D. thermophilus* produced 9.91 ± 0.52 mM of ${\text{NH}}_{3}^{+}$. No nitrite, N_2_, or N_2_O was detected in the logarithmic or stationary phases of growth. The only product of elemental sulfur oxidation was sulfate, and sulfite and thiosulfate were not found in the cultivation medium. In non-inoculated chemical controls the initial nitrate concentration (10 mM) was not changed and no sulfate accumulation was detected during incubation at 65°C for 168 h (**Figure 1C**).

After 48 h of growth, when nitrate was significantly depleted, sulfide could be detected in the cultures, indicating that the cells started to disproportionate elemental sulfur. The total amount of sulfide produced (sum of sulfide in the gas and liquid phases) after 168 h of cultivation was 0.85 ± 0.09 mM for *T. dismutans* and 0.92 ± 0.09 mM for *D. thermophilus*, i.e., ca. 20 times less than the amounts of sulfate produced. Thus, sulfur disproportionation plays a minor role in energy metabolism of both microorganisms in these conditions.

The ratio of sulfate produced to nitrate consumed was 1.55 ± 0.14. This ${\text{SO}}_{4}^{2-}/{\text{NO}}_{3}^{-}$ ratio is close to the theoretical ratio for the reaction:

$$\begin{array}{c} 4{\text{S}}^{0}+3{\text{NO}}_{3}^{-}+7{\text{H}}_{2}\text{O }=\;{4\text{SO}}_{4}^{2-}+3{\text{NH}}_{4}^{+}+2{\text{H}}^{+}\\ \left({\text{reaction}1,[\text{SO}}_{4}^{2-}{/\text{NO}}_{3}^{-}=1:33;\Delta G^{{}^{\circ}\prime }=-325.5\;{\text{kJ mol}}^{-1}{\text{S}}^{0};\Delta G^{{}^{\circ}\prime }=-434.0\;{\text{kJ mol}}^{-1}{\text{NO}}_{3}^{-}\right) \end{array}$$

The greater experimental ratio is probably caused by the production of additional amounts of sulfate during S^0^ disproportionation.

Neither strain grew when hydrogen sulfide (2.0 or 3.5 mM) was added as a potential electron donor instead of elemental sulfur with ${\text{NO}}_{3}^{-}$ as the electron acceptor, or when nitrate was replaced by nitrite (2.5 mM) as a potential electron acceptor with S^0^ as an electron donor. Both microorganisms were capable of sustainable (at least four subsequent 5% v/v transfers) growth either with thiosulfate (10 mM) or sulfite (5 mM) as an electron donor and nitrate (10 mM) as an electron acceptor. Experiments on thiosulfate and sulfite as electron donors for microbial nitrate reduction were hampered due to chemical oxidation of these compounds by nitrate at elevated temperatures. Up to 30% of the thiosulfate was oxidized to elemental sulfur, and up to 40% of the sulfite was oxidized to sulfate in non-inoculated sterile culture medium when incubated with 10 mM KNO_3_ at 65°C for 72 h.

*T. dismutans* and *D. thermophilus* did not grow and did not reduce nitrate when elemental sulfur was entrapped into alginate beads (a nominal molecular mass cutoff of 12 kDa), which prevents the direct contact of microbial cells and insoluble S^0^.

### Genomic Insights into Nitrate Reduction and Sulfur Oxidation

#### Nitrate Reduction

**FIGURE 2:**
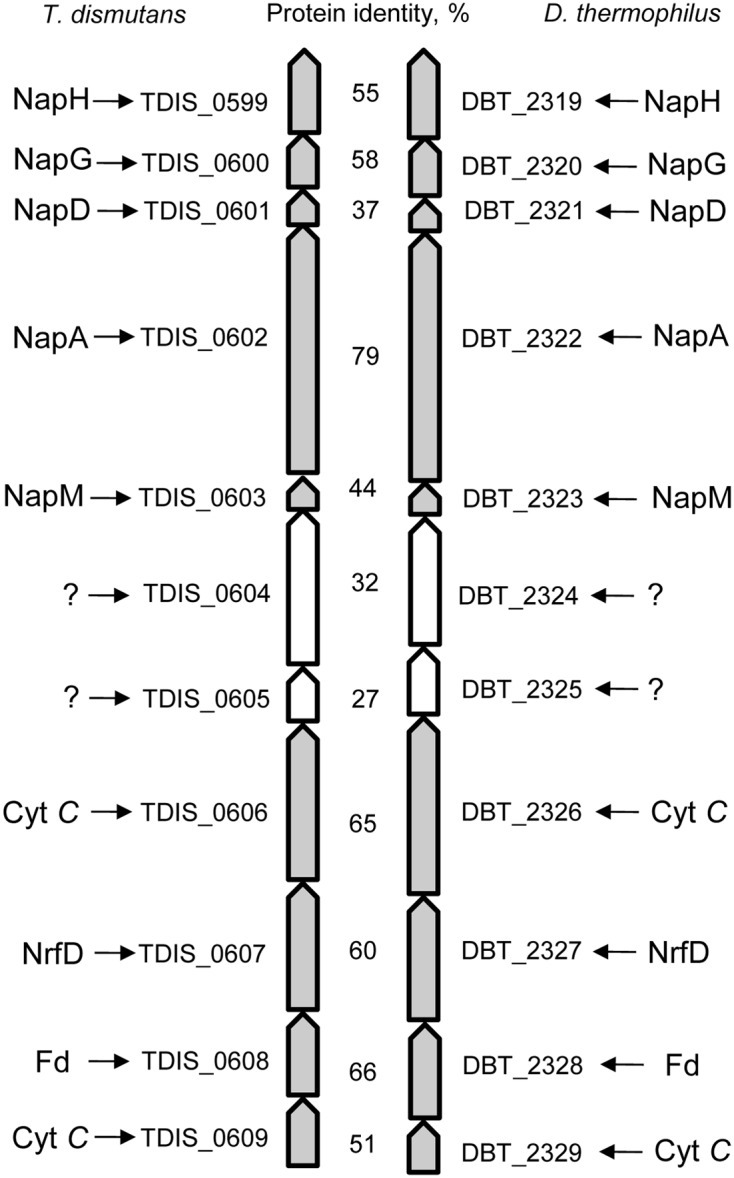
**The 11-gene cluster encoding the Nap-type complex of nitrate reduction and containing other genes relevant to energy metabolism in *T. dismutans* and *D. thermophilus***.

The genomes of *T. dismutans* and *D. thermophilus* contain a similarly organized 11-gene cluster (**Figure 2**). The cluster contains a five-gene locus of nitrate reduction and a four-gene locus relevant to energy metabolism separated by two genes that encode periplasmically oriented proteins of unknown function. In both organisms, nitrate reduction occurs via a Nap-type complex encoded by *napMADGH* (TDIS_0603-0599, DBT_2323-2319). This nitrate reductase complex includes small periplasmic tetraheme cytochrome NapM, the catalytic molybdopterin-binding subunit NapA, cytoplasmic chaperone NapD, and ferredoxin-containing electron transfer subunits NapG and NapH. The Nap system of both bacteria lacks membrane-bound quinol-oxidizing tetraheme cytochrome NapC, which is similar to the nitrate reduction system of epsilon-proteobacteria (Kern and Simon, 2009; Simon and Klotz, 2013). The diheme cytochrome NapB is also absent as in the case of the Nap complex of *Desulfovibrio desulfuricans* (Marietou et al., 2005).

The reduction of produced nitrite to ammonium does not proceed via the canonical Nrf system because the gene homologs of the key enzyme of this pathway, i.e., pentaheme cytochrome *c* nitrite reductase NrfA, are absent in the genomes of both microorganisms. The genomes of *T. dismutans* (Mardanov et al., 2016) and *D. thermophilus* contain genes of octaheme tetrathionate reductase (Otr) (TDIS_1881, DBT_1161) that could be involved in nitrite reduction (Simon et al., 2011). A putative reverse hydroxylamine:ubiquinone reductase module (“reverse HURM”) ammonification pathway has been proposed for epsilon-proteobacterium *Nautilia profundicola* (Campbell et al., 2009; Hanson et al., 2013) but is not completely encoded in the studied organisms, although some genes of this pathway are present. Nevertheless, neither bacteria encode a small subunit of nitrite reductase (membrane-bound tetraheme cytochrome CycB), and the genome of *D. thermophilus* does not contain homologs of Fe-S–containing protein NAMH_1302, which is involved in electron transfer in the cytoplasm. *T. dismutans* and *D. thermophilus* encode cytoplasmic hybrid cluster-containing (HCP) hydroxylamine reductase (TDIS_0472, DBT_1187) as well as several periplasmic or membrane-bound multiheme cytochromes that are homologous to hydroxylamine oxidoreductase (HaoA) of *N. profundicola* (TDIS_0606, TDIS_1483, DBT_0465, DBT_0584, DBT_1609, DBT_1902, and DBT_2326). Ammonium transporters are encoded by the genes TDIS_1605, TDIS_1606, DBT_1335, DBT_1336, and DBT_1339. It can be hypothesized that the four-gene locus (TDIS_0606-0609, DBT_2326-2329) of the 11-gene cluster may be related to nitrite reduction. These four genes encode transmembrane multiheme cytochrome *c*, an integral transmembrane protein of the NrfD family, periplasmic protein with a 4Fe–4S ferredoxin-type iron-sulfur binding domain, and transmembrane cytochrome *c* protein. Alternatively, this locus could be involved in the metabolism of sulfur compounds such as tetrathionate or polysulfides.

#### Sulfur Oxidation

The genomes of *T. dismutans* and *D. thermophilus* did not contain genes of the Sox enzyme system of sulfur oxidation, homologs of sulfur oxygenase reductase (SOR) or genes of sulfide: quinone oxidoreductases (SQRs). The *T. dismutans* genome encodes a complete set of genes required for dissimilatory sulfate reduction (Mardanov et al., 2016). The genome of *D. thermophilus* also contains all the genes of the classical dissimilatory sulfate reduction pathway, including sulfate adenylyltransferase (DBT_0683), manganese-dependent inorganic pyrophosphatase (DBT_1030), APS reductase AprAB (DBT_1479, DBT_1478), the subunits of dissimilatory sulfite reductase DsrABD (DBT_0321, DBT_0322, DBT_0323) and DsrC (DBT_1613), and electron transfer complexes DsrMKJOP (DBT_0287-91) and QmoABC (DBT_1480-82). In both genomes, *dsrC* was located separately from *dsrABD*. The *dsrMKJOP* locus was complemented by two genes that encode DsrN [a co-factor of the glutamine-dependent amidation of siroheme (DBT_0284)] and a small ferredoxin of unknown function (DBT_0283). Like the genome of *T. dismutans*, the genome of *D. thermophilus* contains numerous genes encoding enzymes that may be related to dissimilatory sulfur metabolism, including rhodaneses (DBT_1272, DBT_2204), ferridoxins (DBT_0575, DBT_758, DBT_1900), soluble HdrABC-type heterodisulfide reductases (DBT_0134, DBT_1303, DBT_1305, DBT_1323), and periplasmic molybdopterin oxidoreductases with unknown substrate specificity (tetrathionate, thiosulfate, and polysulfide reductases; DBT_0211, DBT_0827, DBT_1707). The genomes of *T. dismutans* and *D. thermophilus* encode a similar gene cluster comprised of genes that may be involved in elemental sulfur transport and mobilization, i.e., small cytoplasmic sulfur relay proteins DsrE and TusA, and a transmembrane putative transport system permease (TDIS_0918-20, DBT_0019-17).

## Discussion

The results of our study demonstrate the existence of a new pathway of microbial transformation of inorganic compounds – anaerobic oxidation of elemental sulfur by nitrate with the production of ammonium and sulfate. The bacteria performing this process are autotrophic thermophiles that inhabit deep-sea hydrothermal vents, where nitrate ammonification with elemental sulfur may represent a so-far-unrecognized route of primary production. Before this report, only denitrifying microorganisms have been shown to couple sulfur oxidation with nitrate reduction. In deep-sea thermophilic environments, the group of autotrophic elemental sulfur-oxidizing denitrifiers includes *Hydrogenivirga okinawensis* (T opt: 70–75°C), *Persephonella guaymasensis* (T opt: 70°C), *Persephonella marina* (T opt: 73°C), *Piezobacter thermophilus* (T opt: 50°C), and *Thioprofundum lithotrophicum* (T opt: 50°C), (Götz et al., 2002; Nunoura et al., 2008a; Takai et al., 2009). About 10 species of other mesophilic and thermophilic elemental sulfur-oxidizing denitrifiers isolated from various habitats belong to phylum *Aquificae* or the beta, gamma, and epsilon classes of *Proteobacteria* (Huber et al., 1992; Inagaki et al., 2004; Kojima and Fukui, 2010; Slobodkina et al., 2016). The anaerobic oxidation of sulfur compounds coupled to growth has not previously been demonstrated for the members of *Thermodesulfobacteria* or *Deltaproteobacteria*, which were considered exclusively as participants of the reductive branch of the sulfur cycle. The specific growth rates and cell yields observed in our experiments are sufficient to provide substantial biomass accumulation. The reason why microorganisms use ammonification (reaction 1) instead of the more energetically favorable denitrification $\left(5{\text{S}}^{0}+6{\text{NO}}_{3}^{-}+2{\text{H}}_{2}\text{O}=5{\text{SO}}_{4}^{2-}+4{\text{H}}^{+}+3{\text{N}}_{2};\Delta G^{{}^{\circ}\prime }=-548.0\text{ kJ }{\text{mol}}^{-1}\;{\text{S}}^{0};\Delta G^{{}^{\circ}\prime }=-456.7\text{ kJ }{\text{mol}}^{-1}\;{\text{NO}}_{3}^{-}\right)$ is not clear, although it may be explained by the fact that DNRA consumes less nitrate per mol S than denitrification, which could be advantageous in the biotopes with a low nitrate content. Microbial generation time, supply of nitrite relative to nitrate, and the carbon/nitrogen ratio were identified as key environmental controls that determine whether nitrite will be reduced to nitrogenous gas or ammonium in marine microbial nitrate-respiring communities (Kraft et al., 2014). Ammonification dominates over denitrification in electron donor-limiting growth conditions (Yoon et al., 2015). On the other hand, competition for ammonia, the end product of nitrate reduction, influences the structure of chemotrophic microbial communities in geothermal environments (Hamilton et al., 2014).

We found that the direct contact of the cells with solid elemental sulfur was necessary for the growth of *T. dismutans* and *D. thermophilus* during sulfur oxidation. The same effect was observed for a number of sulfur-oxidizing thiobacillii and *Allochromatium vinosum* (Franz et al., 2007 and references therein). In contrast, when *T. dismutans* grows via elemental sulfur disproportionation, the contact of the cells with S^0^ is not required, and most likely soluble polysulfides are the actual substrates in this process (Mardanov et al., 2016). We do not have any plausible explanation for this phenomenon. However, possibly, polysulfides are not chemically stable, and in the presence of high concentrations of nitrate, they are reoxidized to elemental sulfur. In any case, it is evident that the intermediates of elemental sulfur oxidation during nitrate reduction and disproportionation are different.

**FIGURE 3:**
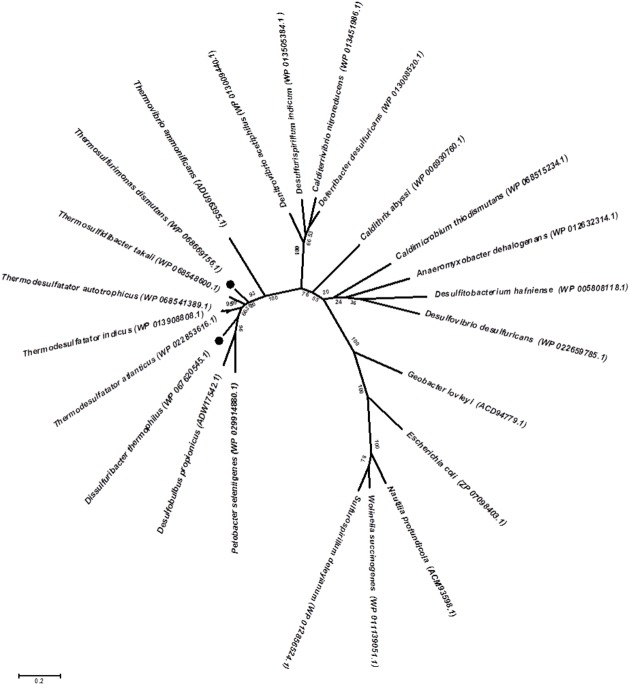
**Maximum likelihood phylogenetic tree showing the position of NapA of *T. dismutans* and *D. thermophilus* within the radiation of bacterial proteins annotated as NapA, the periplasmic nitrate reductases, or molybdopterin-containing oxidoreductases.** Bootstrap values based on 1000 replications are shown at branch nodes. Bar, 2% estimated substitutions.

Genomic insights into energy metabolism show a high level of similarity in the gene pool relevant to nitrogen and sulfur dissimilation in both the studied bacteria and indicate the possible biochemical pathways of nitrate ammonification and elemental sulfur oxidation. Reduction of nitrate to nitrite occurs via a modified Nap-type complex that resembles the nitrate reduction systems of delta- and epsilon-proteobacteria, but has different *nap* gene cluster organization (Simon and Klotz, 2013; Vetriani et al., 2014). Phylogenetic analysis of the catalytic subunit NapA of *T. dismutans* and *D. thermophilus* placed them within a clade with molybdopterin-containing proteins of the members of *Aquificae, Thermodesulfobacteria*, and several *Deltaproteobacteria* (**Figure 3**). NapA of *T. dismutans* and *D. thermophilus* are highly homologous to each other (79% of protein identity) and are closely related to molybdopterin oxidoreductases from *Thermosulfidibacter takaii* (phylum *Aquificae*, 86 and 80% protein identity respectively) and from three species of the genus *Thermodesulfatator* – *T. indicus*, *T. atlanticus*, and *T. autotrophicus* (phylum *Thermodesulfobacteria*, 84 and 81% protein identity respectively). All these microorganisms are deep-sea thermophilic chemolithoautotrophs for which the capacity for nitrate reduction was not reported (Moussard et al., 2004; Nunoura et al., 2008b; Alain et al., 2010; Lai et al., 2016). Further steps of nitrite reduction to ammonium are not evident but both organisms lack pentaheme cytochrome *c* nitrite reductase NrfA. The first option is the participation of octaheme cytochromes in nitrite reduction; these enzymes have broad substrate specificity and can reduce nitrite to ammonium *in vitro* (Atkinson et al., 2007). Otherwise, nitrite reduction might involve hydroxylamine as an intermediate as indicated by the presence of multiheme cytochromes (two in *T. dismutans* and five in *D. thermophilus*), which are homologous to a reversely operating hydroxylamine oxidoreductase that is putatively functioning as a nitrite reductase in *Nautilia profundicola* (Hanson et al., 2013). One of these cytochrome genes is situated in the proximity of the Nap enzyme genes in the 11-gene cluster, which is common for both bacteria (**Figure 2**). This suggests that the location of the nitrate and nitrite reduction systems is in the same cluster. *T. dismutans* and *D. thermophilus* inhabit one and the same hydrothermal site and most likely have acquired the given cluster by lateral gene transfer between members of the microbial community.

Oxidation of elemental sulfur under anaerobic conditions is so far a biochemically unresolved process. Genomes of both microorganisms encode a complete dissimilatory sulfate reduction pathway (Pereira et al., 2011), but genes of conventional sulfur oxidation systems, e.g., bacterial Sox and archaeal SOR (Friedrich et al., 2005; Ghosh and Dam, 2009), are absent. It is possible that in *T. dismutans* and *D. thermophilus*, oxidation of elemental sulfur proceeds via an oxidative DsrAB-type sulfite reductase similar to the intracellular sulfur oxidation in phototrophic sulfur bacterium *Allochromatium vinosum* (Pott and Dahl, 1998). The involvement of the Dsr system is supported by the presence in both bacteria of homologs of genes encoding sulfur relay proteins DsrE, TusA, and Rhd, which could participate in elemental sulfur mobilization (Venceslau et al., 2014). Furthermore, the adenylylsulfate reductase-associated electron transfer complex (Qmo) together with heterodisulfide reductases (Hdr) could also be responsible for elemental sulfur oxidation as was proposed earlier (Quatrini et al., 2009; Finster et al., 2013; Mardanov et al., 2016). The reason why the studied organisms cannot couple growth with sulfate respiration remains enigmatic; the same phenomenon was described for mesophilic sulfur-disproportionating *Desulfocapsa sulfexigens* (Finster et al., 2013). Overall, the genomic data obtained in this study is a challenging task for further transcriptome research.

In conclusion, the results of our study suggest the existence of a novel pathway that couples the sulfur and nitrogen cycles and they demonstrate that the members of *Thermodesulfobacteria* and *Deltaproteobacteria* can participate in the oxidative branch of the sulfur cycle.

## Author Contributions

AS, GS, and EB-O designed the research and wrote the paper; GS, AF, and AM performed the research; AS, NC, and NR analyzed the data.

## Conflict of Interest Statement

The authors declare that the research was conducted in the absence of any commercial or financial relationships that could be construed as a potential conflict of interest.

## Footnotes

1 http://www.cbs.dtu.dk/services/TMHMM/

2 http://www.cbs.dtu.dk/services/SignalP/

3 http://www.compgen.org/tools/PRED-TAT/
